# Experimental Infection of Calves with Transfected Attenuated *Babesia bovis* Expressing the *Rhipicephalus microplus* Bm86 Antigen and eGFP Marker: Preliminary Studies towards a Dual Anti-Tick/Babesia Vaccine

**DOI:** 10.3390/pathogens10020135

**Published:** 2021-01-29

**Authors:** Monica L. Mazuz, Jacob M. Laughery, Benjamin Lebovitz, Daniel Yasur-Landau, Assael Rot, Reginaldo G. Bastos, Nir Edery, Ludmila Fleiderovitz, Maayan Margalit Levi, Carlos E. Suarez

**Affiliations:** 1 Division of Parasitology, Kimron Veterinary Institute, P.O.B. 12, Bet Dagan 50250, Israel; boris@moag.gov.il (B.L.); daniely@moag.gov.il (D.Y.-L.); AsealR@moag.gov.il (A.R.); lu1f@ymail.com (L.F.); maayani.margalit@mail.huji.ac.il (M.M.L.); 2 Department of Veterinary Microbiology and Pathology, College of Veterinary Medicine, Washington State University, Pullman, WA 99164-7040, USA; j.laughery@wsu.edu (J.M.L.); reginaldo_bastos@wsu.edu (R.G.B.); 3 Division of Pathology, Kimron Veterinary Institute, P.O.B. 12, Bet Dagan 50250, Israel; nire@moag.gov.il; 4 Animal Disease Research Unit, Agricultural Research Service, USDA, WSU, Pullman, WA 99164-6630, USA

**Keywords:** *Babesia bovis*, transfected *B. bovis*, anti-*Babesia* vaccine, anti-tick vaccine

## Abstract

Bovine babesiosis, caused by *Babesia bovis* and *B. bigemina*, is a major tick-borne disease of cattle with global economic impact. The disease can be prevented using integrated control measures including attenuated *Babesia* vaccines, babesicidal drugs, and tick control approaches. Vaccination of cattle with the *Rhipicephalus microplus* Bm86-based recombinant vaccine reduces the fitness of *R. microplus* and *R. annulatus*, but several booster inoculations are required to maintain protection. Herein, we generated a stable transfected strain of *B. bovis* expressing an enhanced GFP (eGFP) and a chimeric version of Bm86 (*B. bovis*/Bm86/eGFP). The eGFP was expressed in the parasite cytoplasm, whereas Bm86 was displayed on the surface of merozoites. Three splenectomized calves experimentally infected with *B. bovis*/Bm86/eGFP showed mild signs of acute disease and developed long-lasting antibody responses to *B. bovis* and native Bm86. No evidence of sequestration of parasites in the cerebral capillaries was found upon postmortem analysis, confirming attenuation of the strain. This is the first report of transfected *B. bovis* expressing the tick antigen Bm86 on the merozoite surface that elicits an antibody response to native Bm86. These results represent a proof of concept for a novel live, attenuated, tagged dual-vaccine approach to attempt simultaneous control of babesiosis and tick infestation.

## 1. Introduction

Bovine babesiosis, caused by the intraerythrocytic apicomplexan parasites *Babesia bovis* and *B. bigemina*, is a tick-borne disease of significant economic impact due to the high morbidity and mortality of cattle in tropical and subtropical regions worldwide [1]. *B. bovis* is usually implicated in a severe form of the disease referred to as cerebral babesiosis and similar to cerebral malaria, which is caused by the sequestration of infected red blood cells (RBC) in the brain capillaries, leading to neurological signs and death [1,2]. Ticks of the genus *Rhipicephalus*, primarily *R. annulatus* and *R. microplus*, are the primary competent vectors for the transmission of *B. bovis* and *B. bigemina*, and the presence of these tick populations has been associated with outbreaks of bovine babesiosis in endemic areas [3,4,5,6,7]. 

The most effective method to control acute bovine babesiosis currently available combines approaches of tick management, immunization with attenuated live *Babesia* strains, and the use of anti-*Babesia* drugs [1,3]. Vaccination of less than one-year-old calves with attenuated live *Babesia* parasites remains the main strategy to control and prevent the devastating effects of acute bovine babesiosis in endemic countries, such as Israel, Argentina, and Brazil, among others [1,8,9,10]. Importantly, attenuated *B. bovis* vaccine strains establish persistent infection in cattle and induce strong and long-lasting immunity [1,10,11,12]. In addition, live attenuated *B. bovis* vaccines are generally effective in eliciting protective immunity against heterologous field strains of the parasite [1,10]. However, the currently available live attenuated blood-based vaccines present several drawbacks, such as the risk of transmitting contaminating blood-borne pathogens, the use of several splenectomized calves to produce the attenuated vaccine strains, and potential risks for reversion of virulence [10,13,14]. In addition, some vaccine strains can be acquired and transmitted by ticks in the field, which increases the risks associated with vaccination and poses challenges in discriminating vaccinated from naturally infected animals during outbreaks [6,15].

Currently, tick control mainly relies on the use of acaricides; however, this strategy has severe downsides in food safety and the environment. In addition, ticks eventually develop resistance against most available acaricides. Consequently, alternative approaches such as anti-tick vaccines are urgently needed [16,17,18,19,20]. Ideally, efficient anti-tick vaccines would elicit long-lasting immune responses to decrease tick burden in endemic areas and would prevent the introduction and expansion of ticks into tick-free areas. The most effective anti-tick subunit vaccine developed so far is based on a recombinant version of the concealed midgut glycoprotein antigen Bm86 (recBm86), which was originally identified in *R. microplus* [21,22,23,24,25,26,27,28]. Although Bm86 is originally from *R. micropulus*, recBm86 vaccination protects against infestation from *Rhipicephalus* ticks in general, for instance *R. microplus* and *R. annulatus*, and confers partial protection against ticks of the phylogenetically related genera *Hyalomma* [26,29]. However, Bm86 is a concealed antigen only expressed in the tick midgut and, as such, is not exposed to the bovine immune system during cattle infestation. This implies that “natural” boosters to the primary vaccination cannot be provided during subsequent tick infestations. Thus, as the efficacy of the Bm86 vaccine protection depends on the magnitude and persistence of the anti-Bm86 antibody levels, repeated booster vaccinations are essential for vaccine effectiveness [26,30].

However, despite the need for several booster inoculations of recBm86 emulsified in an adjuvant, this vaccine remains only partially efficient in some field situations and it often requires the use of additional control measures, such as acaricides [26,31,32]. As ticks and tick-borne parasitic diseases become emerging concerns for humans and domestic and wild animals, the development of integrated strategies to control simultaneously ticks and tick-borne parasites are urgently needed. Genetically modified parasites produced by gene manipulation methods have been used to develop novel next generation strategies aimed at controlling vector-borne diseases [33,34,35]. Furthermore, a recent study demonstrated stable incorporation of heterologous genes into the *B. bovis* genome and expression of foreign proteins on the surface of parasite merozoites [36]. This strategy opened the opportunity to use transfected *B. bovis* for the purpose of vaccine development. Additionally, in vivo experiments support the notion of using transfected *B. bovis* parasites as a delivery system to present tick-protective antigens to the bovine immune system and to induce anti-tick immune responses [37]. In that study, the vaccination of bovines with *B. bovis* expressing the glutathione-S-transferase from *Haemaphysalis longicornis* ticks induced specific immune responses against both *Babesia* and the tick antigen, reducing tick fitness [37].

Herein, we expand on these previous concepts by developing and testing in vivo an attenuated strain of *B. bovis* simultaneously expressing an enhanced GFP (eGFP) and the Bm86 antigen of *R. microplus*, termed the *B. bovis*/Bm86/eGFP strain, as a tagged, dual-vaccine candidate against *Babesia* infection and tick infestation. The results demonstrated the expression of Bm86 on the surface of parasite merozoites and that experimental infection of bovines with the attenuated *B. bovis*/Bm86/eGFP strain induced long-lasting specific immune responses against both the tick and *Babesia* antigens. This study provides a strong rationale for developing future evaluations of *B. bovis*/Bm86/eGFP as a live, tagged dual vaccine to simultaneously control tick infestation and acute bovine babesiosis, enabling the differentiation between vaccinated and field strains in cases of *Babesia* outbreaks in vaccinated herds.

## 2. Results

### 2.1. Stable Integration of the R. microplus Bm86 and eGFP Genes into the ef-1α Locus of B. bovis

**Figure 1:**
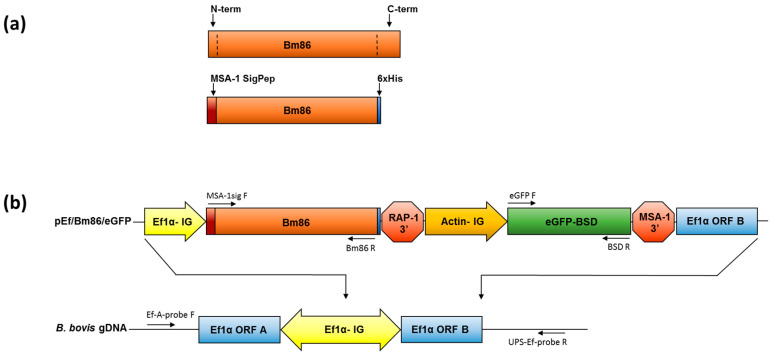
Schematic representation of the *R. microplus Bm86* and plasmid *pEf/Bm86/eGFP*: (**a**) repreScheme 86. (orange box) with the regions encoding for the native N-terminal and C-terminal hydrophobic regions in Bm86. These two regions were removed (dotted lines) to produce the chimeric version of the tick gene (*Bm86Ch*) encoding for the SigPep of the *B. bovis MSA-1* (dark box) and the 6 histidine tag (6xHis) (blue box) minus 100 hydrophobic amino acids at the C terminal end. (**b**) Representation of *pEf/Bm86/eGFP* plasmid containing *Bm86Ch* downstream of the elongation factor 1 (*ef-1α*) intergenic region (IG) and upstream of the *B. bovis RAP-1 3′* regulatory sequence: this plasmid also contains the *eGFP-BSD* ORF cloned downstream of the *B. bovis* actin IG and upstream of the *B. bovis MSA-1 3′*. Additionally, *pEf/Bm86/eGFP* has 680 nucleotides of the *ef-1α* ORF B. Also shown is a representation of the *B. bovis ef-1α* locus where stable integration of *pEf/Bm86/eGFP* is targeted. The locations of the following primers are also shown in the figure: *MSA-1sig F*, *Bm86 R, eGFP F, BSD R, Ef-A-probe F*, and *USP-Ef-probe R.*

In silico secondary structure analysis of the *R. microplus* Bm86 protein sequence revealed the presence of the inserted SigPep of 31 amino acids at its N-terminal (N-term) and a predominantly hydrophobic region with 100 amino acids at its C-terminal (C-term) (Figure S1). Therefore, we generated a synthetic chimeric Bm86 gene, where the sequence encoding for the N-terminal (N-term) SigPep was replaced by the sequence encoding for the *B. bovis* MSA-1 SigPep and the DNA region coding for the 100-amino acid hydrophobic region was replaced by a sequence encoding for a 6xHis tag [36,38] to facilitate detection of the chimera Bm86 protein in transfected parasites. These changes to the original Bm86 gene were designed to facilitate and direct the expression of a modified Bm86 chimera protein, termed Bm86ch, to the surface of transfected *B. bovis* [36], and the detection of parasites expressing the Bm86ch using antibodies against the 6xHis tag. A schematic representation of the resulting chimeric gene is shown in Figure 1a. Bm86Ch was cloned downstream of the intergenic region of the *B. bovis* ef-1α into the transfection plasmid pEf/Bm86/eGFP (Figure 1b). This plasmid also contained the eGFP/BSD fused gene upstream of the 5′-flanking region of *B. bovis* actin, as previously described [39] (Figure 1b). In addition, the transfection plasmid contained a 680 nucleotide fragment representing the 3′-flanking region of ef-1α open reading frame (ORF) B to direct stable integration of the target sequence at the ef-1α locus of the *B. bovis* genome by homologous recombination [33,40], as shown in Figure 1b.

**Figure 2:**
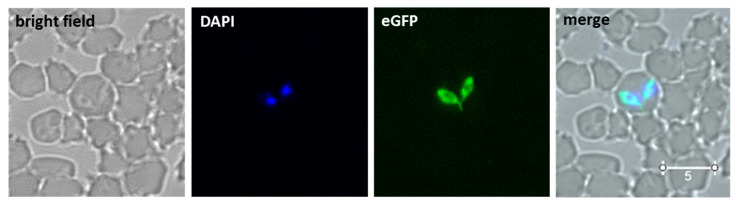
Microscopy analysis of *B. bovis*/Bm86/eGFP: panels show bright field, 4′,6-diamidino-2-phenylindole (DAPI) staining, eGFP fluorescence, and a merged image. The images were taken 14 days after transfection of the parasites growing in blasticidin selection medium. Bar indicates 5 μM.

**Figure 3:**
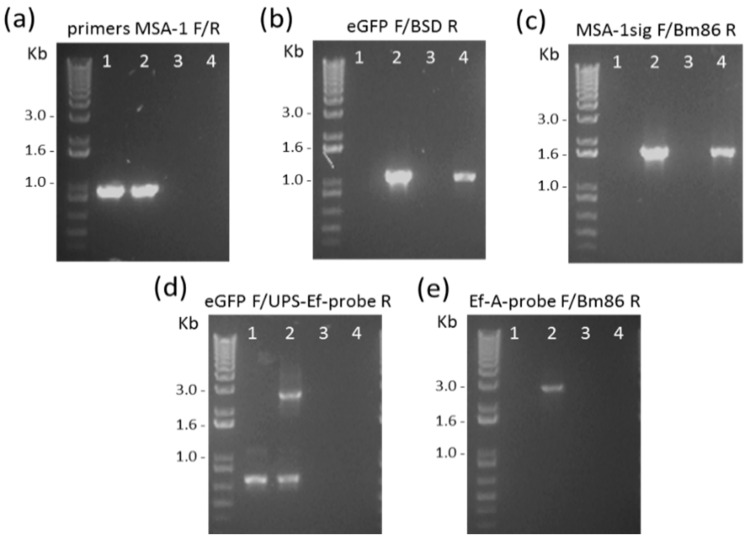
PCR demonstrating the integration of Bm86Ch and eGFP-BSD into the *B. bovis* genome at the expected the ef-1α locus in the parasite line *B. bovis*/Bm86/eGFP: the PCRs were performed using 1) *B. bovis* S74-T3Bo gDNA (wild type strain), 2) *B. bovis*/Bm86/eGFP gDNA, 3) pBluescript plasmid DNA, and 4) pEf/Bm86/eGFP plasmid DNA. The individual panels show the PCR amplicons obtained with primers MSA-1 F/R (**a**), eGFP F/BSD R (**b**), MSA-1sig F/Bm86 R (**c**), eGFP F/UPS-Ef-probe R (**d**), and Ef-A-probe F/Bm86 (**e**).

The plasmid pEf/Bm86/eGFP was transfected into in vitro culture-attenuated *B. bovis* S74-T3Bo parasites that conceivably reduced virulence and tick transmissibility after long-term in vitro culturing. Parasites expressing eGFP in the blasticidin selection medium emerged 14 days after transfection (Figure 2). PCR analysis performed on DNA extracted from the *B. bovis*/Bm86/eGFP parasites demonstrated the expected insertion of Bm86Ch and eGFP-BSD genes into the *B. bovis* ef-1α genome locus (Figure 3). The location of the primers used in the PCRs and a schematic representation of the regions amplified in these experiments are shown in Figure 1b. The PCR results combining the primers for Bm86, MSA-1 SigPep, eGFP, and BSD, and primers EF-A-probe F and UPS-ef-probe R, which recognize sequences located outside the ef-1α locus and absent in the transfection plasmid, demonstrated integration of the target sequences into the ef-1α locus (Figure 3). All PCR amplicons were sequenced to further confirm their identity. Altogether, these data demonstrated that the Bm86Ch and eGFP-BSD genes were specifically integrated by homologous recombination into the *B. bovis* genome at the ef-1α locus of the line *B. bovis*/Bm86/eGFP in a fashion that was fully consistent with our genome insertion strategy.

### 2.2. The R. microplus Bm86Ch is Expressed on the Surface of Transfected B. bovis/Bm86/eGFP

**Figure 4:**
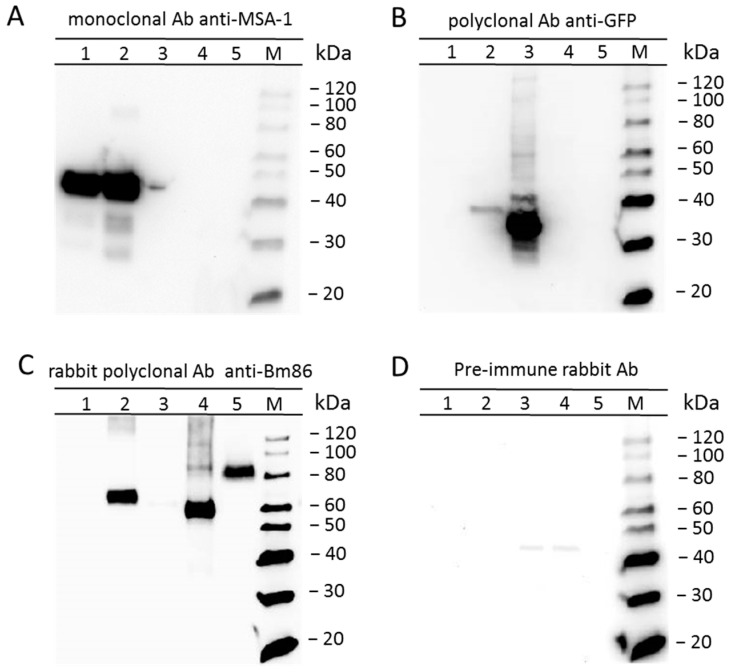
Expression of Bm86Ch and eGFP by *B. bovis*/Bm86/eGFP demonstrated by Western blot analysis: numbers 1 to 5 indicate the lysate of *B. bovis* S74-T3Bo, the lysate of transfected *B. bovis*/Bm86/eGFP, the recombinant eGFP-BSD, the recombinant Bm86, and the *R. microplus* midgut, respectively. (**A**) Monoclonal antibody (Ab) anti-MSA-1 Babb35. (**B**) Rabbit polyclonal Ab anti-GFP. (**C**) Rabbit polyclonal Ab anti-Bm86. (**D**) Pre-immune rabbit serum. The protein molecular weight marker (M) is shown on the right side of each immunoblot.

We first investigated the pattern of expression of Bm86Ch in *B. bovis*/Bm86/eGFP by immunoblotting (Figure 4). Immunoblots using the control monoclonal antibody to *B. bovis* MSA-1 BABB35 demonstrated the expression of a 42 kDa protein in both wild-type parental S74-T3Bo and *B. bovis*/Bm86/eGFP transfected parasites (Figure 4A). In contrast, the expression of eGFP and Bm86 was demonstrated only in lysates of *B. bovis*/Bm86/eGFP-transfected parasites but not in the parental S74-T3Bo strain (Figure 4B,C). Rabbit pre-Bm86 immune serum was used as a negative control for the immunoblot analysis (Figure 4D). The differences in size among the native Bm86 (Figure 4C, lane 5) and the “recombinant” version (Figure 4C, lane 4) are due to the lack of the 100 aa long hydrophobic region and signal peptide that were removed to facilitate the expression of rec Bm86ch in bacteria and in *B. bovis*. However, in contrast to the *E. coli* recombinant Bm86, the *B. bovis* recombinant Bm86ch version also contains the MSA-1 signal peptide and is slightly larger, as is evident in Figure 4C, lane 2.

**Figure 5:**
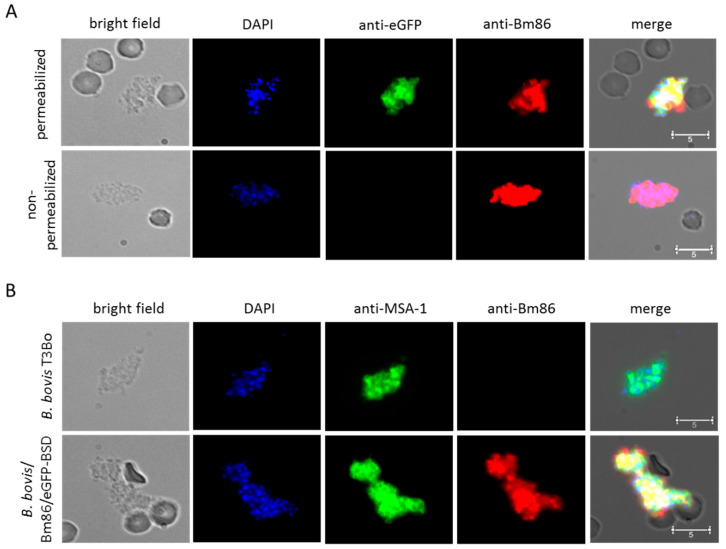
(**A**) Immunofluorescence assay in permeabilized (upper panels) and non-permeabilized (lower panels) *B. bovis*/Bm86/eGFP-transfected parasite merozoites: the panels show bright field and staining with DAPI, anti-eGFP antibodies, and anti-Bm86 antibodies. The scale bar is shown in the bright field panels. (**B**) Immunofluorescence assay in intact, non-permeablilized, extracellular merozoites of the parental S74-T3Bo *B. bovis* (upper panels) and transfected *B. bovis*/Bm86/eGFP parasite line (lower panels): the panels show bright field and staining with DAPI, anti-MSA-1 antibodies, and anti-Bm86 antibodies. The scale bar is shown in the bright field panels. Bar indicates 5 μM.

Next, we determined the cellular localization of Bm86Ch and GFP-BSD in permeabilized and non-permeabilized *B. bovis*/Bm86/eGFP and wild-type control extra-erythrocytic merozoites using immunofluorescence assays (IFAs) (Figure 5). While eGFP expression was only detected in permeabilized parasites, Bm86Ch expression was detected in both permeabilized and non-permeabilized parasites (Figure 5A). These patterns of IFA reactivity are consistent with the expected surface expression of Bm86Ch and the cytoplasmic expression of eGFP. Furthermore, the pattern of Bm86Ch expression on the surface of *B. bovis*/Bm86/eGFP-free merozoites is similar to the pattern of expression of MSA-1, a well-characterized and abundant merozoite surface protein (Figure 5B). We also demonstrated the expression of the Bm86ch antigen in permeabilized intra-intrarythrocytic parasites (Figure S2). Collectively, the IFA data demonstrate the expression of Bm86Ch on the surface of *B. bovis*-free merozoites, providing the rationale for performing in vivo experiments to test the immunogenicity of *B. bovis*/Bm86/eGFP parasites in cattle.

### 2.3. Experimental Infection of Splenectomized Calves with B. bovis/Bm86/eGFP Parasites Elicits Mild Clinical Babesiosis with Production of Antibodies Reactive to Native Bm86

Two splenectomized calves, #89 and #96, were intravenously inoculated with *B. bovis*/Bm86/eGFP parasites, and one additional splenectomized calf (#97) was sub-inoculated intravenously with 50 mL of blood from calf #89 14 days after infection (DPI). All animals were monitored daily for clinical signs of acute babesiosis. A transient period of fever and decrease in packed cell volume (PCV) was observed around 1–2 weeks after inoculation. However, none of the experimentally infected animals required treatment. Animals #89 and #96 developed fever (≥38.8 °C) at 10 DPI, and the additional calf #97 that received the infected blood developed fever starting at 4 DPI. All animals showed drops in PCV that lasted from days 14 to 17 post-inoculation. A low parasitemia ranging from 0.1 to 0.5% was also observed in all inoculated animals. Table 1 summarizes the results of clinical signs of the infected calves. Persistent infection by *B. bovis*/Bm86/eGFP parasites was detected for at least six months by PCR in all infected calves. A positive PCR for Bm86 was also observed in the period of higher parasitemia (two weeks after infection) in all infected calves. After that, a positive PCR to Bm86 was observed only in calf number 97 for at least 67 days. Collectively, the results show that *B. bovis*/Bm86/eGFP-infected calves developed mild symptoms of acute disease, fully consistent with attenuation of the strain, especially considering that all inoculated calves were splenectomized.

**Figure 6:**
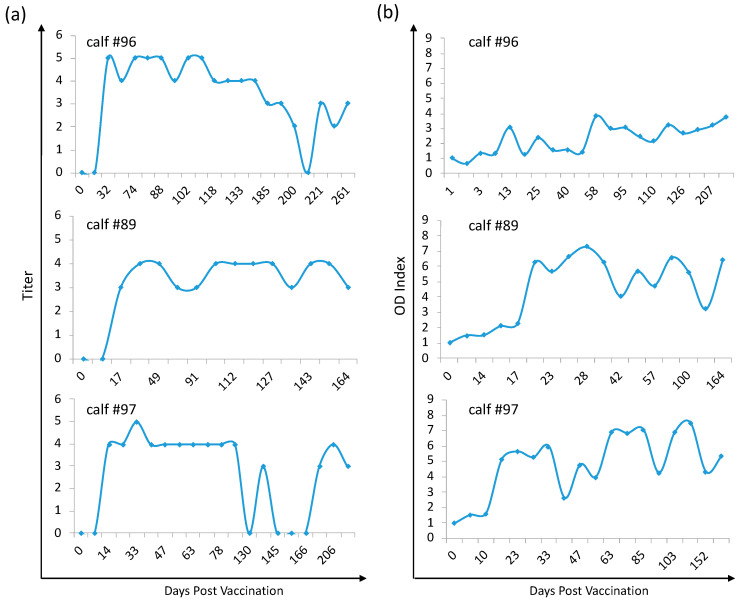
Levels of anti-*B. bovis* (**a**) and anti-Bm86 (**b**) antibodies in *B. bovis*/Bm86/eGFP-infected calves detected by immunofluorescence assay (IFA) and ELISA, respectively: calves #96 and #89 received one intravenous inoculation with 2 × 10^8^ *B. bovis*/Bm86/eGFP-infected red blood cells. Calf #97 was inoculated with 50 mL of blood from calf #96 14 days after prime infection. The results of anti-*B. bovis* serology are presented as a titer where the titration endpoint is reported as the antilog of the serum dilution. The results of anti-Bm86 serology are presented as the optical density (OD) index calculated by the average of the OD of a specific animal sample minus a blank OD divided by the OD of the same animal at time zero minus a blank OD.

All experimentally infected calves developed antibodies against *B. bovis* approximately 10 DPI as determined by IFA with titers ranging from 1:64 to 1:1024 for at least 200 DPI, when the experiment was terminated (Figure 6a and Table S1). Furthermore, infection with *B. bovis*/Bm86/eGFP also induced the production of specific antibodies that react to native Bm86, as shown by ELISA (Figure 6b and Table S2). An increase in the OD index in the Bm86 ELISA test was detected starting approximately two weeks after infection in comparison to the pre-immune samples. Remarkably, increased humoral immune responses to Babesia and Bm86 remained detectable by the ELISA test until the end of the experimental period (Figure 6 and Table S1). Altogether, clinical signs and humoral responses to *B. bovis* and Bm86 indicate that *B. bovis*/Bm86/eGFP was highly immunogenic and established persistent infection in the infected calves.

### 2.4. Fitness Reduction of Ticks Fed B. bovis/Bm86/eGFP-Infected Calves

To start addressing the potential of *B. bovis*/Bm86/eGFP in strategies to control tick infestation, two infected calves (#89 and #97) and one naïve non-infected calves (#99) were infested with 0.3 g of *R. annulatus* larvae. Ticks were put on #89 and #97 calves one and three months after the *B. bovis*/Bm86/eGFP infection, respectively, and allowed to feed and molt to nymphs and adults. The results of the total numbers and weight of engorged females, egg mass, and hatching were evaluated. A reduction of 70% in the number of ticks that fully developed and detached as engorged females was observed. A total of 2587 female ticks fed to repletion in the control calf, whereas 690 and 624 fed to repletion in the two infected animals, respectively (Table 2). The fertility of ticks and the estimated percent of egg hatching did not differ significantly between the infected and control calves. In addition, PCR performed to detect *B. bovis* in ovaries and eggs of female ticks fed infected calves was negative (data not shown).

Overall, despite the low number of calves but considering the ticks as experimental units, the results show a reduction of the fitness of ticks fed *B. bovis*/Bm86/eGFP-infected calves.

### 2.5. Postmortem Examination of Infected Calves

By the end of the experimental period (>200 days post-infection), the animals were humanely euthanized for postmortem analysis of gross lesions and for the presence of Babesia DNA. Considering that PCR and serology demonstrated the establishment of persistent infection by the *B. bovis*/Bm86/eGFP parasite line, it was of interest to investigate signs of pathology in organs of the infected calves, especially evidence for cytoadhesion of infected RBC in the brain capillaries. No gross pathology was found in the postmortem examination and only a single intraerythrocytic parasite was observed adhered to a capillary from the brain of calf #96 (data not shown). No parasites were found in microscopic examinations of smears prepared from the brain, the lungs, spinal cord, kidneys, liver, and heart of infected animals. PCR was positive for *B. bovis* in all tested organs. In addition, PCR for Bm86 was also positive in all organs, except for the kidney and heart of calf #96 and the liver of calf #97 (Table 3). Collectively, these data indicate that *B. bovis*/Bm86/eGFP establishes persistent infection without the development of gross pathology.

## 3. Discussion

Alternative and efficient measures to control both bovine babesiosis and tick infestation are urgently needed to help mitigate the economic burden that these conditions cause in cattle production worldwide. Stably transfected *Babesia* parasites that express GFP during both the acute and persistent phases of the infection have been previously demonstrated [36,41]. Here, we expanded on previous findings by generating transfected parasites that express two independent exogenous proteins, eGFP and the *R. microplus* Bm86 protein, in an attenuated strain of *B. bovis*. It was previously demonstrated that *B. bovis* parasites derived from in vitro culture adapted strains lack virulence [42] and that they cause mild symptoms of acute disease, including small changes in fever and hematocrit in infected cattle. The data in our experiments suggests that the transfected parasites derived from the culture-adapted strain remain attenuated and that it is unlikely that the expression of the Bm86Ch and GFP-BSD exogenous proteins have any additional effects on this phenotype, although this will require further confirmation. Our ultimate goal is to generate genetically modified attenuated *B. bovis* parasites that can serve not only as a vaccine against bovine babesiosis but also as a platform to express protective tick antigens, engendering long-lasting protective immune responses against the parasite and its tick vector. In addition, animals inoculated with such a vaccine could be easily discriminated from non-vaccinated naturally infected animals due to the presence of several markers in the *B. bovis* vaccine strain. Eventually, as novel efficient transfection and gene editing methods are developed, this type of platform could be adapted for expressing other tick antigens in *B. bovis* or other *Babesia* parasites. In this study, we demonstrated that the heterologous genes *eGFP* and *Bm86Ch* were successfully integrated into the *B. bovis* genome at the targeted *ef-1a* locus in a stable fashion, as previously shown by other similar transfection experiments [33,36,37,40]. Furthermore, we showed by in vitro analysis the emergence of eGFP-expressing parasites in a blasticidin selection medium 14 days after transfection. In addition, we demonstrated that the expression of Bm86Ch was efficiently targeted to the surface of free parasite merozoites by using the SigPep of *B. bovis* MSA-1. Interestingly, it appears that, despite the removal of a 100-amino acid region from the Bm86 C-term, which contains a glycosylphosphatidylinositol (GPI) anchor signal, the addition of the *B. bovis* MSA-1 SigPep efficiently directed the expression of this exogenous protein to the surface of merozoites. However, it is possible that the inserted SigPep segment lacks the context needed for cleavage, and as a result, the Bm86Ch protein remains exposed on the surface of the parasite. Consistently, the results show that the merozoite surface expression elicited a humoral immune response to the tick antigen in experimentally infected calves for a significant period after infection. This clearly demonstrates the feasibility of our approach using transfected *B. bovis* as a vaccine platform to deliver tick antigens.

Stable integration of the *Bm86Ch* and *eGFP* genes in the *B. bovis* genome and heterologous expression of these proteins offer a unique opportunity that can significantly improve the current live attenuated *B. bovis* vaccines that are routinely used for the control of bovine babesiosis. For instance, it is currently cumbersome to distinguish vaccine strains and field isolates of parasites during outbreaks in vaccinated herds [6,15]. Here, we demonstrate the expression of eGFP by blood stages of the parasite, as previously described [36]. Therefore, eGFP expression can potentially serve as a marker to differentiate between vaccine and field strains, which can have an important impact on the epidemiology of bovine babesiosis in endemic areas where vaccination with live parasites is routinely used.

Vaccines based on recBm86 have been shown to be an efficient strategy to reduce infestation of *R. micropulus* and *R. annulatus* ticks, although inconsistent results have been observed in field tests in distinct geographic locations [26]. Ticks fed on recBm86-vaccinated cattle show a decreased number of engorged females, lighter weight, and a decreased reproductive capacity compared to ticks fed on non-vaccinated animals [22,27]. It has also been shown that cattle vaccinated with recBm86 develop strong antibody responses and that the vaccine efficacy ranges from 51% to 99% in reducing tick infestation under experimental conditions [23,26,29]. Here, we demonstrate that calves infected with *B. bovis*/Bm86/eGFP developed a significant level of antibodies that recognize native Bm86. Considering that *B. bovis*/Bm86/eGFP infection elicited antibodies that recognize native Bm86, we then started addressing the potential of this genetically modified parasite to control tick infestation. We observed an approximately 70% reduction in the number of engorged females fed on *B. bovis*/Bm86/eGFP-infected calves, compared to ticks fed on a non-infected calf. In addition, PCR analyses of the ovaries and eggs of engorged females fed on vaccinated animals were negative to the presence of *B. bovis*, suggesting that the *Babesia*-transfected parasites appear to be not transovarial transmitted. Nevertheless, we did not examine the occurrence of transstadial transmission by checking different ticks stages or salivary glands. Thus, the possible transmission of transfected parasites by ticks should be further evaluated. Although limited by the small number of immunized animals, these results clearly show a reduction in the total number of engorged ticks on *B. bovis*/Bm86/eGFP-infected calves compared to ticks fed on the control animal.

The route of administration should also be taken in consideration when comparing the *B. bovis*/Bm86/eGFP and recBm86-based formulations. In our approach, both parasite and tick antigens are continuously presented to the cattle immune system by the transfected *B. bovis*/Bm86/eGFP parasites; as such, the exact amount of antigen involved cannot be estimated. In contrast, a known amount of recombinant antigen emulsified in the adjuvant is inoculated when using the recBm86 vaccination protocol, which involves several boost inoculations per animal [24,25,26]. Also, the use of adjuvants can bias the type of immune responses elicited to an identical or similar antigen [43]. Thus, it is possible that different mechanisms of antigen presentation could possibly lead to the production of different types of functionally distinct antibodies, in terms of class, affinity, and specificity, according to the vaccine platform. In addition, Bm86Ch expressed by transfected *B. bovis* may be structurally different compared to the recombinant protein produced either in eukaryotic or prokaryotic expression systems. Further studies using a larger number of animals are needed to address these aspects.

A high correlation between the level of antibody response and vaccine efficacy was observed in cattle immunized with the recBm86 vaccine [23]. Therefore, vaccination boosters at 4 and 7 weeks post-prime immunization and revaccination every six months are essential to maintain the levels of antibodies in vaccinated animals [23]. In this study, persistent titers of anti-Bm86 antibodies were detected for 9 months after a single infection with *B. bovis*/Bm86/eGFP. Thus, the data demonstrate that a single infection with the transfected parasites was sufficient to elicit long-lasting antibody production. Persistent infection of the transfected parasites was demonstrated by PCR in different organs by postmortem examination despite the continuous elicitation of anti-Bm86 and anti-*B. bovis* antibodies in infected calves. This finding suggests that these antibodies do not affect the establishment of persistent infection, which is a hallmark feature of *Babesia* parasites. It was beyond the scope of the study to compare the *B. bovis*/Bm86/eGFP parasites to the parental *B. bovis* strain S74-T3Bo in vivo; however, the clinical parameters and the lack of parasite sequestration in the splenectomized experimentally infected animals suggest that the transfected line has an attenuated phenotype. Therefore, collectively, our results demonstrate both attenuation and the ability of the transfected parasites to establish persistent infection, as previously demonstrated with similarly transfected parasites derived from the *B. bovis* Mo7 strain [41]. In addition, considering the small number of calves used in our study, *B. bovis*/Bm86/eGFP-infected animals were not challenged with a virulent *B. bovis* strain. However, our results establish a solid foundation and strong rationale for developing additional studies to further evaluate attenuated strains of *B. bovis* to express protective tick antigens as a dual vaccine against bovine babesiosis and tick infestation.

## 4. Materials and Methods

### 4.1. B. bovis Parasites

The *B. bovis* S74-T3Bo strain used in this study [44] was maintained in an in vitro culture for long-term passages (LTp) (>300 passages). As a definition for this study, one passage was counted every time that the culture split, and fresh medium and RBC were added to the culture. A typical *B. bovis* culture was passaged every 48 h. Parasites were maintained in microaerophilous stationary-phase culture using 10% of bovine RBC in HL-1 medium (Sigma-Aldrich, St. Louis, MO, USA) supplemented with 40% bovine serum at 37 °C and 5% CO_2_, as previously described [45]. The LTp parasites were used for genetic manipulation experiments using a stable transfection system, as previously described [36,46].

### 4.2. Construction of Transfection Plasmid

A synthetic DNA sequence was constructed containing a full-length *Bm86* gene (GenBank accession number M29321) [21] minus the sequences encoding for the N-term signal peptide (31 amino acids) and the predominantly hydrophobic domain at its C-term (100 amino acids). The synthetic *Bm86* gene also contained the sequences encoding for the *B. bovis MSA-1* signal peptide (SigPep,) at N-term and a 6xHis tag at its C-term. *Bm86Ch* was placed downstream of the *B. bovis ef-1α* intergenic region and upstream of the *B. bovis* RAP-1 3′ to control the termination of gene transcription (Figure 1A,B). In addition, the *eGFP* gene fused with the blasticidin S deaminase gene (*BSD*) (*eGFP/BSD*) [40] was placed downstream of the *B. bovis* actin intergenic region [39] and upstream of the *B. bovis* MSA-1 3′ regulatory sequence (Figure S2B). The *Bm86Ch* and *eGFP/BDS* genes in addition to the regulatory sequences and the 5′ and 3′ *ef-1α* insertion sequences were placed directionally into the *Sac*I and *Kpn*I sites of the pBluescript plasmid, resulting in the plasmid pEf/Bm86/eGFP (Figure 1B). All synthetic DNA sequences were produced by GenScript.

### 4.3. B. bovis Transfection

Twenty micrograms of pEf/Bm86/eGFP or control pBlueScript plasmids were diluted in 25 μL cytomix buffer (120 mM KCl, 0.15 mM CaCl_2_, 10 mM K_2_HPO_4_/KH_2_PO_4_ pH 7.6, 25 mM HEPES, 2 mM EGTA, and 5 mM MgCl_2_, final pH 7.6) and electroporated with 75 μL of approximately 20% *B. bovis* infected bovine red blood cells (RBC), as previously described [36,40]. Six hours after transfection, blasticidin (4 μg/mL) (Thermo Fisher Scientifics, Waltham, MA, USA) was added to the parasite cultures. The culture medium, containing blasticidin, was replaced daily, and the cells were monitored by fluorescent microscopy for the emergence of eGFP-expressing parasites.

### 4.4. Analysis of B. bovis/Bm86/eGFP Parasites

The *B. bovis*/Bm86/eGFP line and parental parasites were expanded in culture to 25% parasitized RBC and then harvested for genomic DNA (gDNA) isolation. Twenty nanograms of gDNA from *B. bovis*/Bm86/eGFP and parental parasites in addition to 5 ng of pBlueScript and pEf/Bm86/eGFP plasmids were used for PCR with the following primers: eGFP-F (atggtgagcggcgaggagctgttc) and BSD-R (gccctcccacacataaccagagggcagc), MSA-1sig-F (gcctagggatccgcggccgcatg), Bm86-R (gacactgcgattctgctttatgg), and eGFP-F and UPS-Ef-probe-R (cacgcgcaatatcacagttccatc). The primers MSA-1-F (gatgcgtttgcacatgctaag) and MSA-1-R (cgggtacttcggtgctctca) were also used as *B. bovis* housekeeping controls. The locations of the primers are shown in Figure 1B. The PCR products were sequenced to verify the integration of *Bm86Ch* and *eGFP/BSD* ORFs into the *B. bovis ef-1α* locus.

### 4.5. Immunoblotting

Cell lysates of the *B. bovis*/Bm86/eGFP and parental parasites, recombinant eGFP-BSD, recBm86, and *R. microplus* midgut were analyzed by immunoblotting. Four replicates of each set of samples were subjected to SDS-PAGE and transferred to nitrocellulose membranes. Membranes were blocked in 5% milk and incubated separately using specific primary and secondary antibodies. Monoclonal antibody anti-MSA-1 (BABB35) [47] (20 μg/mL), anti-eGFP polyclonal antibodies (Invitrogen) (1/1000 dilution), anti-Bm86 rabbit polyclonal antibodies (1/500 dilution) [36], and pre-immune polyclonal rabbit serum (1/500 dilution) [36] were used as primary antibody reagents. Goat anti-mouse IgG peroxidase conjugate (Life Biosciences, Boston, MA, USA) and goat anti-rabbit IgG peroxidase conjugate (Life Biosciences, Boston, MA, USA) were used following the manufacture’s protocol. The immune complexes were revealed using an enhanced chemiluminescence method (ECL™) (Amersham).

### 4.6. Indirect Immunofluorescence Assays

Extraerythrocytic free merozoites were isolated from expanded *B. bovis*/Bm86/eGFP and parental parasites using centrifugation, as previously described [36]. The samples were then washed in 3% bovine serum albumin (BSA) and aliquoted for use in cell permeabilized and non-permeabilized indirect immunofluorescence assays (IFA). For permeabilized IFA, samples were first smeared on a slide, fixed for 5 min in 100% acetone, and then incubated with 0.1% Triton X-100. The slides were then incubated in 10% BSA for 1 h with a combination of anti-MSA-1 antibodies (2 ug/mL) and anti-Bm86 antibodies (1/500), or anti-eGFP antibodies (2 ug/mL) and anti-Bm86 antibodies (1/500). The slides were washed three times with PBS and incubated in 10% BSA with goat anti-rabbit IgG Alexa Fluor^®^ 555 and goat anti-mouse IgG Alexa Fluor^®^ 488. The slides were then washed three times with PBS and mounted with a drop of Prolong™ Gold Anti-fade with 4′,6-diamidino-2-phenylindole(DAPI) (Thermo Fisher, Waltham, MA, USA) and cover slip. Non-permeabilized samples were incubated with the antibodies, washed within a 1.5 mL tube, then fixed on a slide, and mounted in an identical manner as the permeabilized samples. The slides were analyzed using a Leica SP8-X White Light Laser point scanning confocal microscope (Leica Microsystems, Wetzlar, Germany). The digital images were processed using Leica LAS X analysis software (Leica Microsystems, Wetzlar, Germany) to produce individual and merged images.

### 4.7. Experimental Infection of Calves with B. bovis/Bm86/eGFP

Three Holstein dairy calves derived from a non-grazing dairy herd and serologically negative for both *Babesia* and Bm86 were used for experimental infection with *B. bovis*/Bm86/eGFP. The animals were kept in a closed barn to exclude the possibility of natural tick infestation throughout the experiment. Two splenectomized calves (#89 and #97), 4 months of age, were infected intravenously with 2 × 10^8^ *B. bovis*/Bm86/eGFP-infected RBCs. A third splenectomized calf (#96) was infected with 50 mL of blood from calf #89 when parasitemia was approximately 0.005% and the PCV was 24 (25% drop) at 14 days after infection. One uninfected calf (#99), also 4 months of age, was used as a control. All animals were monitored daily for fever, PCV, and parasitemia. Serum samples were collected from each animal once a week to evaluate the development of a humoral immune response to *B. bovis* and Bm86 antigens. Peripheral blood was also collected from the animals and used for PCR to detect *B. bovis* and Bm86 DNA.

### 4.8. Serology

Serum samples were collected from the peripheral blood of each experimental animal and kept at −20 °C until use. Specific antibodies to *B. bovis* were assessed by IFA, as previously described [48]. The samples were four-fold diluted (from 1:16 to 1:1024). Fluorescence with serum dilution at 1:64 and above was considered positive based on the available known controls. The results of *B. bovis* serology are presented as a titer, where the titration endpoint is reported as the reciprocal of the serum dilution. The presence of specific antibodies against Bm86 in the sera of infected calves was determined by ELISA. Briefly, the Bm86 antigen was prepared by dissecting the *R. annulatus* tick midgut. Individual midguts were placed in a 2 mL tube with lysis buffer containing a protease inhibitor (Sigma-Aldrich, St. Louis, MO, USA). The tubes were then frozen at −20 °C overnight. After that, homogenized tick midgut lysates were sonicated three times for 30 s followed by incubation on ice for one minute. After sonication, the samples were incubated for 5 min at 95 °C and centrifuged at 14,000× *g* for 3 min. The amount of protein in the supernatants was measured, and the samples were stored for −20 °C until use. The Bm86 antigen was used to coat ELISA plates overnight at 4 °C at the concentration of 10 µg/mL in a coating buffer (BioLegend, San Diego, CA, USA). The plates were washed 3 times with PBS 0.05% tween 20 (PBS-T) for 5 min and blocked with skim milk 2% in PBS-T for 1 h at 37 °C. The plates were then washed as described above. Serum samples in triplicates were diluted 1:10 in PBS-T, and 100 uL was added per well. The plates were incubated for 1 h at 37 °C and then washed again as described above. Secondary antibody sheep anti-bovine IgG HRP-conjugated (Bethyl laboratories, Montgomery, Ala, USA) (1:50,000) was added to individual wells, and the plates were incubated for 1 h at 37 °C. The reaction was developed using the 1-Step™ Ultra TMB-ELISA Substrate Solution (Thermo Fisher Scientific, Waltham, MA, USA), and the plates were read at OD 450 nm. The results of anti-Bm86 serology are presented as semiquantitative data, where the OD index was calculated by the following formula: OD index = (average OD sample in a specific date)/(average OD sample pre-immunization). The samples were normalized by subtracting the average of the OD blank values, and an OD index ≥ 1 was considered positive.

### 4.9. PCR Assays

Genomic DNA from the blood, brain, lungs, spine cord, kidneys, liver and, heart of infected calves was extracted using the Maxwell^®^ 16 Blood and tissue DNA Purification Kit (Promega, Wisconsin, WI, USA) and kept in −20 °C until use. For the detection of *B. bovis* DNA, a nested PCR was used as previously described [48]. For the detection of *Bm86*, the following primers were used: Bm86-hphil-F (tcatccatttgctctgacttcgggaaa) and Bm86-hphil-R (gacactgcgattctgctttatgg). PCR for *Bm86* was performed as follows: 95 °C for 3 min, followed by 35 cycles, each consisting of denaturation at 95 °C for 30 s, annealing at 56 °C for 30 s, and extension at 72 °C for 1 min. An additional final extension step at 72 °C for 5 min was included.

### 4.10. R. annulatus Infestation

*Babesia*-free *R. annulatus* larvae (0.3 g/calf) were used to infest the *B. bovis*/Bm86/eGFP-BSD-infected and control calves. Calves #96 and #97 were infested with *R. annulatus* one and three months after infection, respectively. Engorged tick females started dropping from the calves from days 21 to 28 of feeding. The dropped engorged females were collected, washed in water, counted into groups of 30 ticks, and placed in Petri dishes at 28 °C and 85% relative humidity. Approximately 48 h after dropping, the tick females were weighed, and their survival was assessed during the following three weeks after dropping. After that, oviposition, hatching, egg mass weigh, and eggs fertility were recorded. Fertility of ticks were analyzed by ratios of egg mass weight/female weight. To estimate the percent of hatching, eggs taken from all engorged ticks from each calf were placed on top of the Whatman filter paper in a Petri dish at 25 °C and 85% relative humidity. After hatching ended, the egg mass was homogenized and observed in a stereoscope microscope. The proportion of estimated hatched of eggs was measured after 19–40 days using binocular microscope for each sample. The estimated rate of hatching was divided in 5 categories: 1 = 1–20%, 2 = 21–40%, 3 = 41–60%, 4 = 61–80%, and 5 = 81–100%. The means of estimated hatching of each sample were the percentage of hatching, as previously described [49]. The presence of *B. bovis* DNA in tick eggs was examined by PCR as described above. At the end of the experiment, nine months after *B. bovis*/Bm86/eGFP-BSD infection, all calves were humanely euthanized and submitted to postmortem examination, according to the requirements of the Kimron Veterinary Institute (KVI) Animal Welfare Committee.

## 5. Conclusions

Here, we present an attenuated, eGFP-tagged *B. bovis* strain as a platform to express protective tick antigens, for instance, Bm86, for a potential dual-vaccine approach to control *Babesia* infection and tick infestation. The advantages of such vaccine strategy include (a) the expression of tick antigens by *B. bovis* as an eukaryotic system rather than by prokaryotic organisms; (b) the constitutive expression and delivery of tick antigens by the parasite during persistent infection, which results in enhanced antigen presentation to the cattle immune system and maintenance of high antibody titers; (c) the proposed vaccine implying a single inoculation strategy, considering the establishment of persistent infection; (d) a reduction in vaccination costs due to simplification of the practical aspects related to vaccine production and the potential single-vaccination approach of cattle in the field; (e) an alternative approach to controlling tick infestation, with this vaccine reducing the use of toxic and environmentally unsafe acaricides and decreasing the risk of tick populations developing acaricide resistance; and (f) the inclusion of the molecular marker eGFP into the vaccine strain for identification and differentiation of vaccinated from naturally infected animals in the field. Collectively, it can be anticipated that an effective transfected vaccine strain of *Babesia*-expressing protective tick antigens would have strong practical and economic impacts on the control of bovine babesiosis and tick infestation.

## Figures and Tables

**Table 1:** Clinical signs of calves infected with *B. bovis*/Bm86/eGFP parasites.

Calves	Inoculum (Dose/Route)	Fever (≥38.8 °C)		PCV		Parasitemia	
		Start (DPI)	Duration (days)	Highest Drop (%)	Duration (days)	Highest PPE (%)	Duration (DPI)
#89	*B. bovis*/Bm86/eGFP (2 × 10^8^ parasites/IV)	10	6	28.1	17	0.1	10–17
#96	*B. bovis*/Bm86/eGFP (2 × 10^8^ parasites/IV)	10	5	25	14	0.5	10–15
#97	Blood from calf #89 (50 mL/IV)	4	6	40.6	17	0.5	4–10

DPI: Days post-infection. PCV: Packed cell volume. PPE: Percent of parasitized erythrocytes.

**Table 2:** Fitness parameters of ticks fed *B. bovis*/Bm86/eGFP-infected calves.

	*B. bovis*/Bm86/eGFP		Control
Calf Number	#96	#97	#99
Total number of engorged ticks	690	624	2587
Mean tick weight (g ± SD)	0.26 ± 0.05	0.26 ± 0.03	0.27 ± 0.04
Mean egg mass (g ± SD)	0.13 ± 0.03	0.14 ± 0.02	0.14 ± 0.06
Eggs weight/female weight (g ± SD)	0.49 ± 0.06	0.53 ± 0.03	0.49 ± 0.06
Rate of egg hatching * (E ± SD)	4.31 ± 0.68	4.32 ± 0.73	4.30 ± 0.64

(*): Estimated rate of egg hatching (E) was divided in 5 categories: 1 = 1–20%, 2 = 21–40%, 3 = 41–60%, 4 = 61–80%, and 5 = 81–100%. SD: represents standard deviation. No statistical significance was found (*p* < 0.05).

**Table 3:** PCR results for *B. bovis* and *Bm86* of the postmortem evaluation of *B. bovis*/Bm86/eGFP-infected calves. (+) and (−) represent positive and negative PCR reaction respectively.

PCR	*B. bovis*		*Bm86*	
Calf Number	#96	#97	#96	#97
Brain	+	+	+	+
Lungs	+	+	+	+
Spine cord	+	+	+	+
Kidney	+	+	−	+
Liver	+	+	+	−
Heart	+	+	−	+

## Data Availability

Data is contained within the article or Supplementary Material.

## Supplementary Materials

The following are available online at https://www.mdpi.com/2076-0817/10/2/135/s1, Figure S1: (a) Native nucleotide sequence of the *Rhipicephalus microplus Bm86* (GenBank accession number M29321). (b) Chimeric Bm86 (Bm86Ch) containing the *B. bovis* MSA-1 signal peptide at its N-terminal (blue font) and the 6 His tag at its C-terminal (blue font); Figure S2: Permeabilized immunofluorescence assay using *B. bovis*/Bm86/eGFP-infected erythrocytes stained with A) DAPI, anti-eGFP, and anti-Bm86 and B) DAPI, anti-eGFP, and anti-MSA1 to produce a merged image; Table S1: Titer of antibodies by immunofluorescence antibody test (IFAT ) Table S2: ELISA summary data including mean OD, standard deviation, and index calculation.
