# Supplementary material for: Experimental Infection of Calves with Transfected Attenuated Babesia bovis Expressing the Rhipicephalus microplus Bm86 Antigen and eGFP Marker: Preliminary Studies towards a Dual Anti-Tick/Babesia Vaccine
Source: Pathogens. 2021 Jan 29;10(2):135. doi: 10.3390/pathogens10020135 (PMC7911397; doi:10.3390/pathogens10020135)
Supplement: Supplementary file 1 [file pathogens-10-00135-s001.zip › supp materials/Fig S2.pptx]

## Slide 1
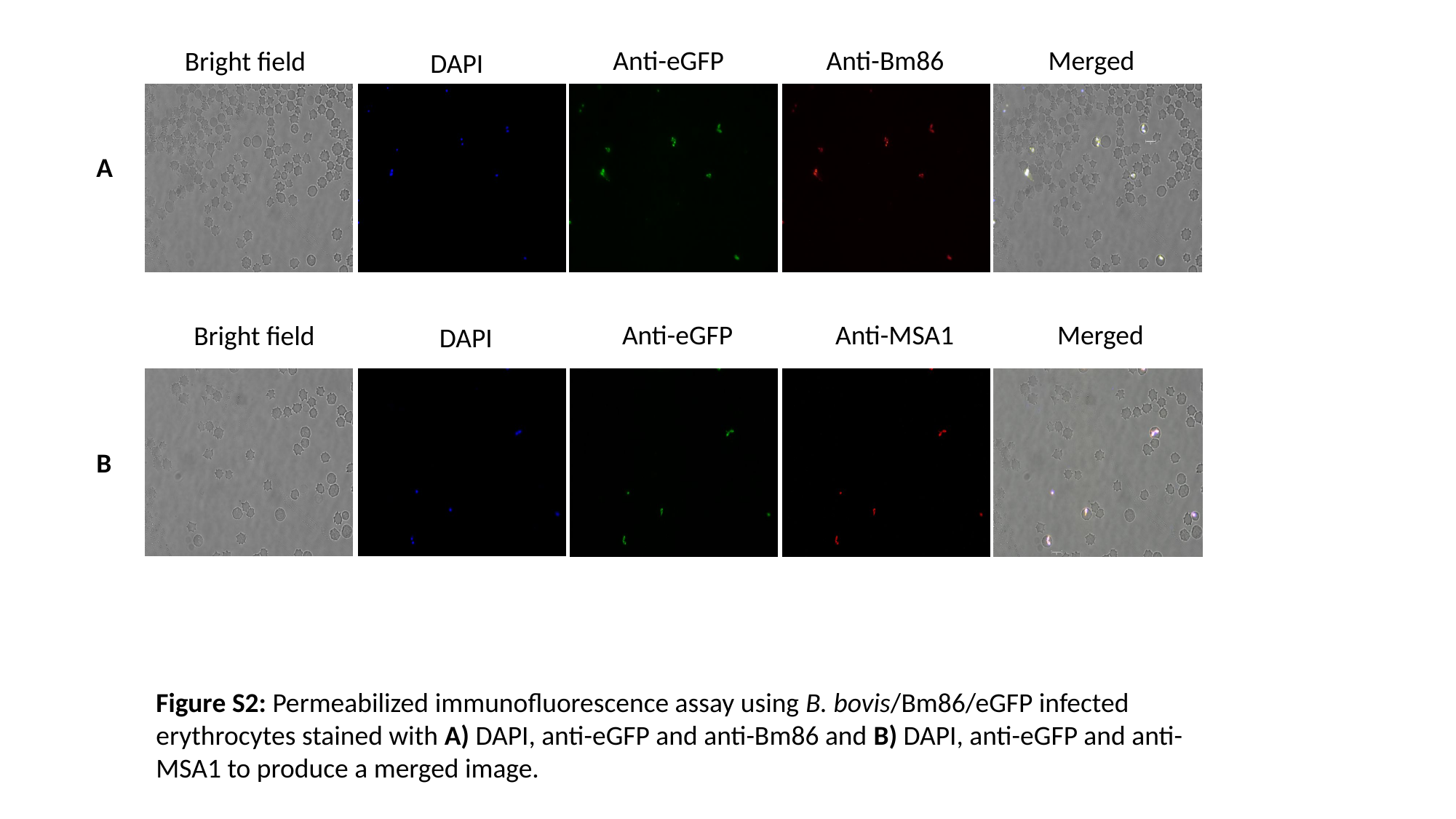

Anti-eGFP
Anti-Bm86
Merged
Bright field
DAPI
A
Anti-eGFP
Anti-MSA1
Merged
Bright field
DAPI
B
Figure S2: Permeabilized immunofluorescence assay using B. bovis/Bm86/eGFP infected erythrocytes stained with A) DAPI, anti-eGFP and anti-Bm86 and B) DAPI, anti-eGFP and anti-MSA1 to produce a merged image.
